# Alleviation of Saline–Alkaline Stress in Alfalfa by a Consortium of Plant-Growth-Promoting Rhizobacteria

**DOI:** 10.3390/plants14172744

**Published:** 2025-09-02

**Authors:** Lingjuan Han, Yixuan Li, Zheng Ma, Bin Li, Yinping Liang, Peng Gao, Xiang Zhao

**Affiliations:** 1 College of Grassland Science, Shanxi Agricultural University, Jinzhong 030801, China; hanlj@sxau.edu.cn (L.H.); 20233295@stu.sxau.edu.cn (Y.L.); z20223243@stu.sxau.edu.cn (Z.M.); liangyinping@sxau.edu.cn (Y.L.); gaopeng@sxau.edu.cn (P.G.); 2 College of Horticulture, Shanxi Agricultural University, Jinzhong 030801, China; libin080@sxau.edu.cn

**Keywords:** saline–alkali stress, bacterial consortium, alfalfa, growth, soil fertility

## Abstract

Soil salinization critically threatens global agricultural productivity by impairing plant growth and soil fertility. This study investigated the potential of a consortium, comprising *Acinetobacter calcoaceticus* DP25, *Staphylococcus epidermidis* DP28, and *Enterobacter hormaechei* DP29, to enhance the saline–alkali tolerance of alfalfa and improve soil properties. The experiments comprised five germination treatments (saline control, each strain alone, consortium) and three pot treatments (non-saline control, saline control, consortium). Under saline–alkali stress, co-inoculation with the consortium significantly (*p* < 0.05) increased alfalfa seed germination rates, emergence rates, and biomass (shoot and root dry weight), while promoting root development. Physiological analyses revealed that the bacterial consortium mitigated stress-induced damage by enhancing photosynthetic efficiency, chlorophyll content, and antioxidant enzyme activities (superoxide dismutase (SOD), peroxidase (POD), and catalase (CAT)), while decreasing malondialdehyde (MDA) levels. Moreover, the inoculant improved osmoprotectant accumulation (soluble sugars, soluble proteins, and proline) and modulated soil properties by reducing pH and electrical conductivity (EC), while elevating nutrient availability and soil enzyme activities. Correlation and principal component analyses (PCA) confirmed strong associations among improved plant growth, physiological traits, and soil health. These findings demonstrate that the bacterial consortium effectively alleviates saline–alkali stress in alfalfa by improving soil health, offering a sustainable strategy for ecological restoration and improving agricultural productivity in saline–alkali regions.

## 1. Introduction

Soil salinization represents a critical ecological and environmental challenge worldwide, jeopardizing food security and sustainable agricultural by lowering crop yields and diminished soil fertility [1]. Sodium carbonates (Na_2_CO_3_ and NaHCO_3_), accumulated in arid and semiarid regions within saline–alkali soils, are more toxic to plants than NaCl. Beyond the osmotic imbalance, ion toxicity, and oxidative damage typically induced by salinity, alkali stress further imposes high-pH conditions that disrupt intracellular pH homeostasis, compromise membrane integrity, and decrease both root vitality and photosynthetic function [2,3]. Moreover, saline–alkali stress reduces soil microbial diversity and enzyme activity, impedes nutrient cycling, and significantly inhibits plant growth [4].

Employing microbial inoculants for the bioremediation of saline–alkali land provides an environmentally friendly strategy that supports sustainable and cost-efficient agriculture [5,6]. Among these, plant-growth-promoting rhizobacteria (PGPR) are widely used and contribute to plant growth and nutrient acquisition under abiotic stress through diverse mechanisms. The key mechanisms include (1) producing phytohormones (especially indole-3-acetic acid (IAA)) and secreting 1-aminocyclopropane-l-carboxylic acid (ACC) deaminase that reduces the precursor of ethylene; (2) enhancing nutrient availability (such as that of iron (Fe), nitrogen (N), phosphorous (P), and potassium (K)) through the production of siderophores, nitrogenase, and organic and sugar acids, respectively; and (3) secreting exopolysaccharide (EPS) during microbial activities, which significantly enhances rhizosphere soil aggregation to improve plant stress tolerance [7,8,9]. Additionally, PGPR induce systemic resistance and promote signal transduction, as well as photosynthesis rates, thereby indirectly increasing plant growth [10]. While numerous microorganisms show potential in mitigating soil salinity, single-strain inoculant continue to demonstrate limited performance due to their inability to simultaneously adapt to the multifactorial challenges of soil pH, osmotic pressure, organic matter availability, and salinity levels. In contrast, bacterial consortia exhibit superior remediation efficiency through synergistic interactions among constituent strains [11]. These interactions often involve mutual growth stimulation and functional integration, such as cooperative biofilm formation and shared substrate metabolism, which facilitate biogeochemical cycling, as well as improve agricultural productivity and environmental remediation applications [12].

Alfalfa (*Medicago sativa* L.), known as the “King of Forages”, possesses both high nutritional quality and considerable economic importance. With moderate saline–alkali tolerance, it is widely cultivated in saline–alkaline regions for soil remediation and improvement [13,14]. However, saline–alkali stress continues to limit alfalfa production, primarily by reducing germination rates, slowing growth, and reducing yields [15,16,17]. In our previous study, we found that *Acinetobacter calcoaceticus* DP25, *Staphylococcus epidermidis* DP28, and *Enterobacter hormaechei* DP29 originally isolated from the rhizosphere soil of *Lespedeza daurica* exhibited multiple promoting growth properties (PGPs) and strong salt–alkali resistance. Therefore, the objectives of this study were to study the effects of co-inoculation with these three strains on alfalfa in saline–alkali environments through (1) analyzing seedling emergence and growth, (2) investigating physiological responses, and (3) evaluating soil physicochemical properties. The findings are expected to provide theoretical support for the combined application of microbial inoculants in alfalfa cultivation within saline–alkali environments.

## 2. Results

### 2.1. Effects of Bacterial Consortium on Alfalfa Seed Germination Under Saline–Alkali Stress

As shown in Table 1, compared with CK, all inoculation treatments significantly affected alfalfa seed germination parameters under saline–alkali stress. Among them, co-inoculation treatments exhibited stronger promoting effects than single-inoculation treatments, increasing germination rate, germination potential, germination index, and vigor index by 8.14–16.85, 27.99–51.77%, 56.60–70.12%, and 14.42–16.13%, respectively. Based on these parameter analyses, we selected the most effective treatment (co-inoculation) for subsequent experiments.

### 2.2. Effects of Bacterial Consortium on Emergence Rate and Growth of Alfalfa Under Saline–Alkali Stress

**Figure 1:**
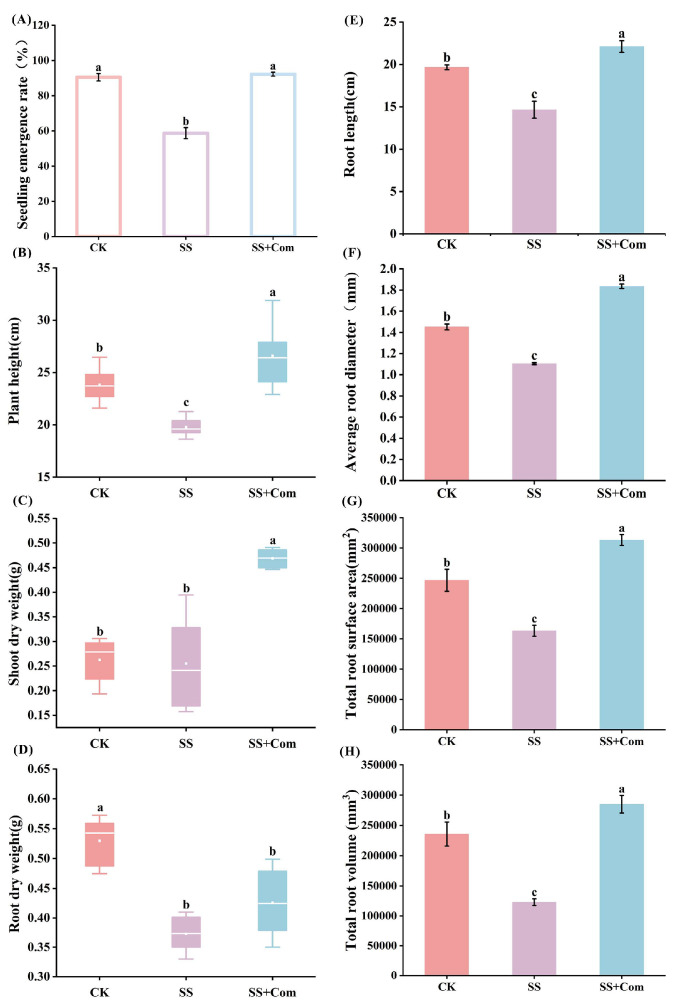
Effects of bacterial consortium on emergence rate and growth of alfalfa under saline–alkali stress. (**A**) Seedling emergence rate, (**B**) plant height, (**C**) shoot dry weight, (**D**) root dry weight, (**E**) root length, (**F**) average root diameter, (**G**) total root surface area, (**H**) total root volume. CK: no saline–alkali stress; SS: saline–alkali stress; SS + Com: saline–alkali stress + combination. Different letters indicate significant differences (*p* < 0.05).

Saline–alkali stress remarkably inhibited the emergence rate and growth of alfalfa compared with CK (Figure 1). However, under saline–alkali conditions, the plants treated with co-inoculation significantly increased in seedling emergence rate, plant height, and shoot dry weight by 57.02%, 34.34% and 80.77%, respectively, compared with those in the SS treatment (Figure 1A–C). Further, co-inoculation treatments noticeably enhanced the root morphological parameters of alfalfa seedlings under saline–alkali stress compared to SS treatment (Figure 1E–G). Overall, co-inoculated PGPR enhanced plant emergence rate and alleviated the inhibitory effects of saline–alkali stress on alfalfa growth.

### 2.3. Effects of Bacterial Consortium on Photosynthetic Parameters and Photosynthetic Pigments of Alfalfa Under Saline–Alkali Stress

**Figure 2:**
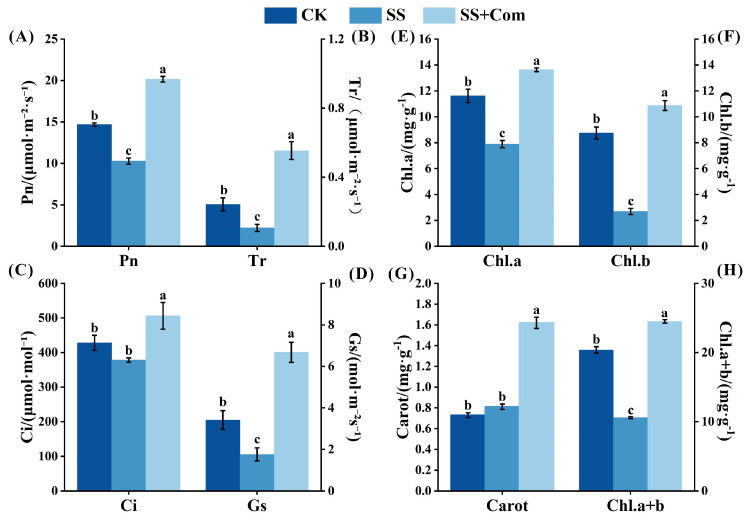
Effects of bacterial consortium on photosynthetic parameters and photosynthetic pigments of alfalfa under saline–alkali stress. (**A**) Net photosynthetic rate (Pn), (**B**) transpiration rate (Tr), (**C**) intercellular CO_2_ concentration (Ci), (**D**) stomatal conductivity (Gs), (**E**) chlorophyll a (Chl. a), (**F**) chlorophyll b (Chl. b), (**G**) carotenoid (Carot), (**H**) total chlorophyll (Chl. a + b). CK: no saline–alkali stress; SS: saline–alkali stress; SS + Com: saline–alkali stress + combination. Different letters indicate significant differences (*p* < 0.05).

Saline–alkali stress negatively impacted photosynthetic parameters and photosynthetic pigments of alfalfa (Figure 2). However, co-inoculation decreased the inhibitory effect of saline alkalization on Pn, Tr, Gs, Chl a, Chl b, and total chlorophyll content. Compared with the non-inoculated treatment under stressed conditions, co-inoculated treatment significantly improved those parameters by 0.96-, 4.00-, 2.79-, 0.73-, 3.04-, and 1.32-fold, respectively.

### 2.4. Effects of Bacterial Consortium on Antioxidant Enzyme Activities and MDA of Alfalfa Under Saline–Alkali Stress

**Figure 3:**
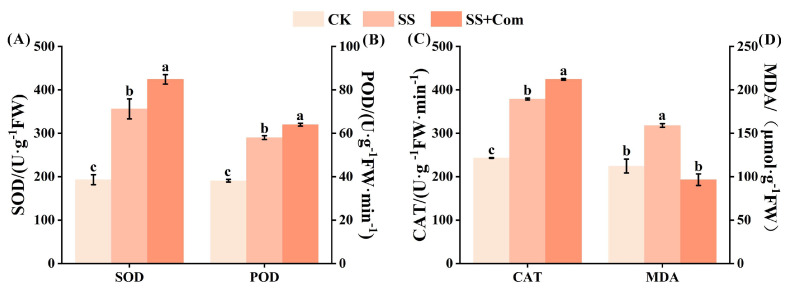
Effects of bacterial consortium on antioxidant enzyme activities and MDA of alfalfa under saline–alkali stress. (**A**) Superoxide dismutase (SOD), (**B**) peroxidase (POD), (**C**) catalase (CAT), (**D**) malondialdehyde (MDA). CK: no saline–alkali stress; SS: saline–alkali stress; SS + Com: saline–alkali stress + combination. Different letters indicate significant differences (*p* < 0.05).

Figure 3 illustrates the changes in SOD, POD, CAT, and MDA contents in response to saline–alkali stress. Alfalfa seedlings in the SS treatment exhibited notably increased production of SOD, POD, CAT, and MDA in their leaves as compared to the plants in the CK treatment. A remarkable increase was observed in SOD, POD, and CAT levels due to co-inoculated PGPR; these increases were 19.12%, 10.23%, and 12.00%, respectively. However, the MDA content in the co-inoculated treatment was noticeably decreased by 39.30% under saline–alkali conditions compared to SS treatment.

### 2.5. Effects of Bacterial Consortium on Osmoprotectant Levels of Alfalfa Under Saline–Alkali Stress

**Figure 4:**
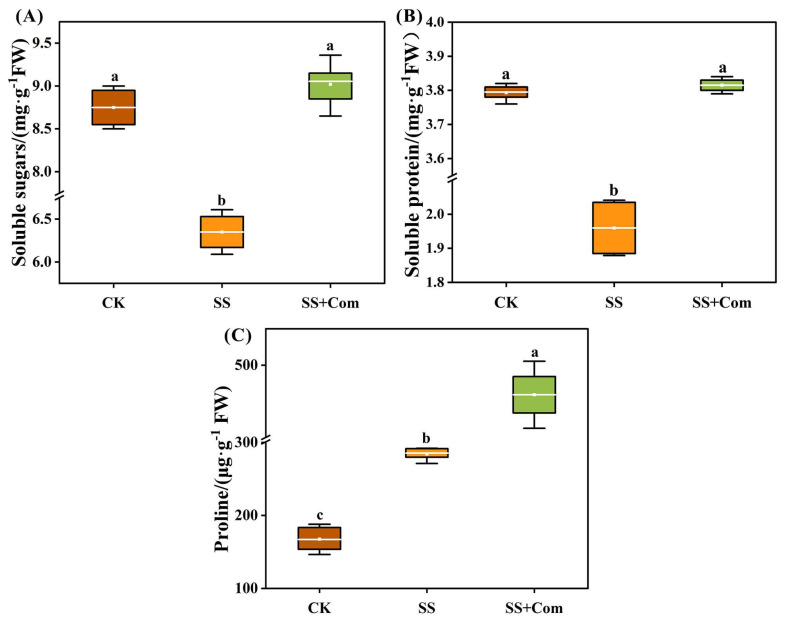
Effects of bacterial consortium on osmoprotectant levels of alfalfa under saline–alkali stress. (**A**) Soluble sugars, (**B**) soluble protein, (**C**) proline. CK: no saline–alkali stress; SS: saline–alkali stress; SS + Com: saline–alkali stress + combination. Different letters indicate significant differences (*p* < 0.05).

The effects of saline–alkali stress on alfalfa seedlings significantly led to increased proline (Pro) content and decreased contents of soluble sugars (SSs) and soluble protein (SP) compared to unstressed control plants (CKs) (Figure 4). However, treatment with co-inoculated PGPR positively improved these parameters. Compared to SS treatment, the contents of SS, SP, and Pro were noticeably increased by 42.05%, 94.90%, and 61.60%, respectively, in co-inoculated treatment.

### 2.6. Effects of Bacterial Consortium on Rhizosphere Soil Physicochemical Properties

**Figure 5:**
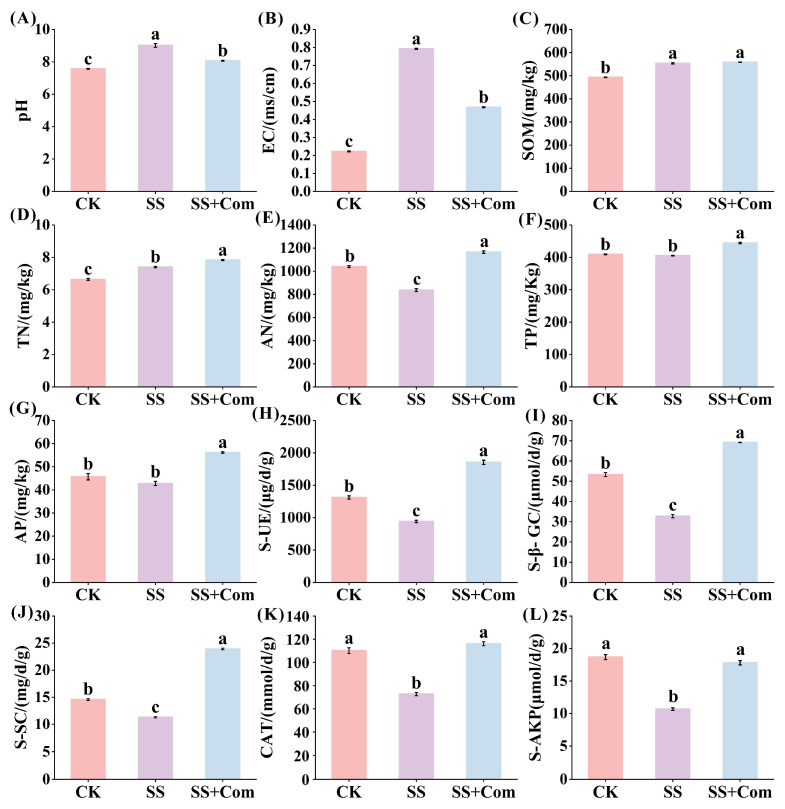
Effects of bacterial consortium on rhizosphere soil physicochemical properties. (**A**) Soil pH (pH), (**B**) electrical conductivity (EC), (**C**) soil organic matter (SOM), (**D**) total nitrogen (TN), (**E**) available nitrogen (AN), (**F**) total phosphorus (TP), (**G**) available phosphorus (AP), (**H**) soil urease (S-UE), (**I**) soil β-glucosidase (S-β-GC), (**J**) soil sucrase (S-SC), (**K**) soil catalase (CAT), (**L**) soil alkaline phosphatase (S-AKP). CK: no saline–alkali stress; SS: saline–alkali stress; SS + Com: saline–alkali stress + combination. Different letters indicate significant differences (*p* < 0.05).

Co-inoculated treatments greatly altered the physicochemical properties of alfalfa rhizosphere soil under stressed conditions (Figure 5). Compared to the control (CK), saline–alkali stress significantly increased soil pH and electrical conductivity (EC) (Figure 5A,B). However, the plants with co-inoculated PGPR showed pronounced lower soil pH and EC as compared to plants without PGPR inoculation under saline–alkali stress. In addition, microbial inoculation modified soil nutrient contents (Figure 5C–G). The co-inoculation treatment significantly increased soil total nitrogen (TN), available nitrogen (AN), total phosphorus (TP) and available phosphorus (AP) in the alfalfa rhizosphere under saline–alkali conditions. Regarding soil enzyme activity, saline–alkali stress resulted in a significant reduction in the activity of soil urease (S-UE), β-glucosidase (S-β-GC), sucrase (S-SC), catalase (CAT), and alkaline phosphatase (S-AKP) compared to CK (Figure 5H–L). However, compared with the SS treatment, the co-inoculation treatment had a positive effect on these contents, with increases of 0.97-, 1.11-, 1.12-, 0.60-, and 0.75-fold, respectively.

### 2.7. Correlation Analysis Between Growth Parameters and Physicochemical Properties

**Figure 6:**
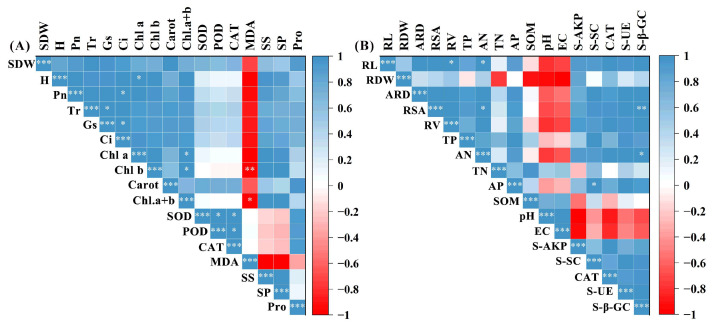
Correlation analysis between growth parameters and physicochemical properties. (**A**) Aboveground growth parameters and plant physicochemical properties (SDW, shoot dry weight; H, plant height; Pn, net photosynthetic rate; Tr, transpiration rate; Gs, intercellular CO_2_ concentration; Ci, stomatal conductivity; Chl a, chlorophyll a; Chl b, chlorophyll b; Carot, carotenoid; Chl a + b, total chlorophyll; SOD, superoxide dismutase; POD, peroxidase; CAT, catalase; MDA, malondialdehyde; SS, soluble sugars; SP, soluble protein; Pro, proline). (**B**) Root growth parameters and soil physicochemical properties (RL, root length; RDW, root dry weight; ARD, average root diameter; RSA, total root surface area; RV, total volume of roots; TN, total nitrogen; AN, available nitrogen; TP, total phosphorus; AP, available phosphorus; SOM, soil organic matter; pH, soil pH; EC, electrical conductivity; S-AKP, soil alkaline phosphatase; S-SC, soil sucrase; CAT, soil catalase; S-UE, soil urease; S-β-GC, soil β-glucosidase). “*”, *p* < 0.05; “**”, *p* < 0.01; “***”, *p* < 0.001.

The results of Pearson correlation analysis between the plant growth parameters and physiological and biochemical indicators in the microbial treatments under stress conditions are exhibited in Figure 6. In the aboveground parameters, plant height and SDW were positively correlated with the photosynthetic parameters, photosynthetic pigments, and osmoprotectant levels, whereas a negative correlation was observed with MDA (Figure 6A). In the underground parameters, a positive correlation was observed between the root indicators (length, average diameter, total surface area, and volume) and soil physicochemical parameters (TP, TN, AP, AN, and soil enzyme activity), whereas a negative correlation was observed with soil pH and EC (Figure 6B).

### 2.8. Principal Component Analysis (PCA) Between Soil Nutrients and Soil Enzyme Activities

**Figure 7:**
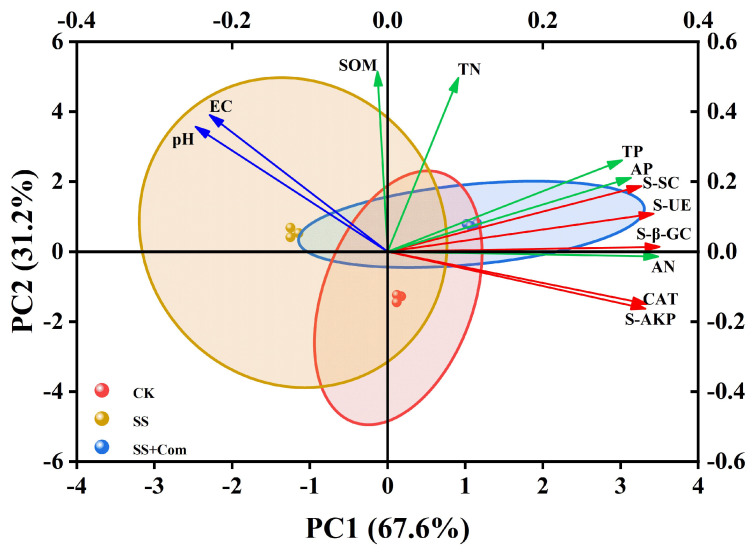
Principal component analysis (PCA) of soil nutrients and soil enzyme activities. TN, total nitrogen; AN, available nitrogen; TP, total phosphorus; AP, available phosphorus; SOM, soil organic matter; pH, soil pH; EC, electrical conductivity; S-AKP, soil alkaline phosphatase; S-SC, soil sucrase; CAT, soil catalase; S-UE, soil urease; S-β-GC, soil β-glucosidase. CK: no saline–alkali stress; SS: saline–alkali stress; SS + Com: saline–alkali stress + combination.

To understand the relationships among the results for inoculation microbes, soil nutrients, and soil enzyme, multivariate principal component analysis (PCA) was performed (Figure 7). The first two principal components (PCs) explained 98.8% of the observed cumulative variance, with 67.6% and 31.2% in the first (PC1) and second (PC2) principal components, respectively. The PCA showed a significant intergroup difference among all treatments, indicating dissimilar responses from each other. PC1 was found to be positively associated with TP, AP, AN, and soil enzyme. PC2 was found to be positively associated with TN and SOM. These variables were strongly associated with the treatment of plants inoculated with PGPR.

## 3. Discussion

Soil salinization, characterized by ionic toxicity and oxidative stress, exerts strong inhibitory effects on plant growth and development. Plant-growth-promoting rhizobacteria (PGPR) are known to alleviate abiotic stresses by enhancing nutrient acquisition, photosynthesis, and antioxidant capacity. Our results demonstrated that PGPR inoculation significantly improved alfalfa seed germination and vigor index under saline–alkali stress, a finding that is consistent with earlier reports. Bal et al. [18] observed that ACC deaminase-producing PGPR increased IAA levels in seeds, stimulating α-amylase activity and accelerating starch hydrolysis to release soluble sugars for germination. Moreover, the three-strain consortium in our study produced stronger beneficial effects than single-strain treatments, highlighting the advantages of a multi-microbe approach. This is consistent with previous studies showing that a microbial consortium generally provides greater adaptability and biological activity than single-strain inoculants [19,20]. In addition, in our study, the bacterial consortium was shown to significantly enhance seedling emergence, biomass content (shoot and root dry weight), and root development, effectively alleviating the growth inhibition caused by saline–alkali stress. Khoso et al. [1] observed that inoculation with a salt-tolerant PGPR enhanced alfalfa root growth and stress tolerance by modulating endogenous hormone signaling and antioxidant defenses. Similarly, it is likely that metabolites from our bacterial strains helped regulate the host’s phytohormone balance, thereby promoting cell division in root meristems and improving overall plant vigor under stress.

Photosynthesis is one of the particularly stress-sensitive physiological processes in plants. Generally, under saline conditions, plants often show declines in chlorophyll content and photosynthetic rate, together with impaired photosystem activity and disruption of the electron transport chain [21]. In the present study, the adverse effect of saline–alkali stress on photosynthetic parameters and photosynthetic pigments were obvious in alfalfa seedlings (Figure 2). By contrast, multi-strain PGPR inoculation substantially improved these traits under stress conditions. Chen et al. [22] likewise reported that PGPR treatment increased chlorophyll concentration and photosynthetic efficiency in rice under salt stress. One probable mechanism is that PGPR facilitate nutrient uptake (for instance, providing more Mg^2+^ for chlorophyll synthesis) and improve leaf water status, thereby delaying stress-induced chlorophyll degradation [23,24]. In addition, photosystem II (PSII) is particularly vulnerable to oxidative damage induced by reactive oxygen species (ROS), leading to reduced photosynthetic efficiency. Antioxidant enzymes (SOD, POD, and CAT) can effectively eliminate excess ROS and help maintain redox balance, thereby supporting plant performance. Malondialdehyde (MDA), a marker of lipid peroxidation, reflects membrane stability and the degree of oxidative injury. In our study, PGPR-inoculated alfalfa leaves showed significantly higher SOD, POD, and CAT activities, coupled with lower MDA content, compared to non-inoculated plants, indicating reduced oxidative damage. Li et al. [25] observed a similar trend in maize, where PGPR inoculation significantly decreased leaf MDA under saline–alkali conditions by boosting the plant’s antioxidant defense system. Furthermore, Pearson correlation analysis further supported these results, revealing positive associations between growth performance and photosynthetic parameters, chlorophyll content, and antioxidant enzyme activity, while MDA was negatively correlated (Figure 6A). These results indicate that PGPR sustain photosynthetic function and overall plant performance under saline–alkali stress by enhancing the host’s antioxidant capacity, which scavenges excess ROS and protects photosystem structures.

Osmotic regulators (such as soluble sugars (SS), soluble proteins (SP), and proline (Pro)), critical biochemical indicators of plant stress resistance, can help sustain cellular osmotic balance and limit dehydration under stress. In addition, Pro, acting as a chemical chaperone to stabilize protein structures, enhances enzyme activities and scavenges reactive oxygen species (ROS). Xian et al. [26] reported that seedlings under saline–alkali stress showed increased levels of Pro, SS, and SP, whereas, in our study, saline–alkali stress significantly reduced SS and SP but elevated Pro content (Figure 4). This discrepancy may be attributed to saline–alkaline stress inhibiting photosynthesis and carbon assimilation, thereby limiting SS synthesis. Previous studies have shown that approximately 80% of the CO_2_ assimilated during photosynthesis was converted into SS [27]. Moreover, this process also induces protease activity, accelerating SP degradation and providing precursor substrates for Pro synthesis [28,29,30]. Many studies have shown that PGPR modulate osmolyte metabolism under stress, thereby enhancing plant tolerance [31,32,33]. Similarly, our results showed that multi-strain inoculation (DP25, DP28, and DP29) improved osmolyte profiles under saline–alkali stress, maintaining higher SS and SP while further enhancing Pro accumulation. Moreover, a strong positive correlation between plant growth indices (SDW and plant height) and osmoprotectant contents was observed through Pearson correlation analysis (Figure 6A). Therefore, these results indicated that our multiple strains (DP25, DP28, and DP29) are beneficial to maintain the normal osmotic pressure of alfalfa seedlings and improve the adaptability of alfalfa plants to saline–alkali stress.

Using PGPR is recognized as an effective approach to ameliorate soil nutrient status and promote plant performance under saline–alkali conditions. Soil pH and EC, as critical indicators of soil health, strongly influence nutrient availability and enzyme activity. In this experiment, PGPR inoculation led to reduced pH and EC under saline–alkali stress. This may be attributed to organic acid secretion by PGPR, which neutralizes alkaline compounds in the soil and lowers pH. Studies have further demonstrated that PGPR can absorb soil salts and either transform them into growth nutrients or metabolize them into less harmful forms, thus lowering EC [34,35]. Our findings are in line with these reports and further supported by PCA analysis, which revealed negative correlations between soil nutrient levels and both pH and EC (Figure 7). In addition, the activities of soil enzymes play essential roles in modulating fertility and responding rapidly to environmental changes. Among soil enzymes, urease catalyzes the hydrolysis of urea into plant-available nitrogen, while β-glucosidase, a key enzyme mediating the decomposition and transformation of organic matter, is widely used as a sensitive soil quality index linked to carbon cycling [36,37]. Soil catalase, sucrase, and alkaline phosphatase contribute to nutrient (C, N, P, K) release through carbohydrate hydrolysis and organic matter mineralization [38]. Our results clearly showed that PGPR inoculation significantly enhanced soil enzymatic activities under saline–alkali stress, which correlated with improved nutrient availability and was positively associated with root morphological traits (length, diameter, surface area, and volume) (Figure 5, Figure 6 and Figure 7). These results align with previous findings that PGPR promote root growth by stimulating soil enzyme activity and nutrient cycling, thereby alleviating the inhibitory effects of environmental stress [39].

## 4. Materials and Methods

### 4.1. Preparation of Microbial Inoculant

DP25 (*Acinetobacter calcoaceticus*), DP28 (*Staphylococcus epidermidis*), and DP29 (*Enterobacter hormaechei*) were originally obtained from the rhizosphere soil of *Lespedeza daurica* in the Taigu experimental field (China) and are currently maintained at the College of Grassland Science, Shanxi Agricultural University. Each strain was initiated from a single colony and cultured in LB broth at 28 °C with shaking (150 rpm) for 24 h to generate bacterial suspensions. Bacterial cells were harvested by centrifugation (5000 rpm for 10 min, 4 °C) and resuspended in sterilized water to an OD600 of 0.8, corresponding to approximately 1.0 × 10^9^ CFU/mL. The suspensions of the 3 strains were mixed in equal volumes (*v*/*v*) to formulate a bacterial consortium [40]. Prior to mixing, potential antagonistic interactions were evaluated by plate confrontation assays, and no inhibitory effects were detected [41].

### 4.2. Seed Inoculation

The alfalfa seed (“Zhongmu No.3”) was purchased from Beijing Rytway Seed Co., Ltd. (Beijing, China), and subsequently coated with the inoculum of the three bacterial strains. Briefly, according to the percentage of seed weight, the seeds were mixed and coated with 30% film-forming agent (polyvinyl alcohol (PVA)–water-soluble chitosan (CS) = 9:1, *w*/*w*), 5% water-retaining agent (superabsorbent polymer, SAP), 45% filler (diatomaceous earth–bentonite = 9:1, *w*/*w*), trace elements (ZnSO_4_, 0.02%; FeSO_4_, 0.04%), 1% coloring agent (carmine red), and 10% of the above bacterial consortium solution. Before coating, seeds were surface-sterilized by washing with sterile water, soaking in 0.5% sodium hypochlorite for 15 min, and rinsing three times with sterile water.

### 4.3. Seed Germination Experiment Design

Healthy and uniform alfalfa seeds were surface-sterilized by washing with sterile water, immersing in 0.5% sodium hypochlorite for 15 min, rinsing three times with sterile water, and air-drying. The sterilized seeds were then soaked in the bacterial suspension for 10 h, while control seeds were treated with sterile water, followed by air-drying. Treated seeds were germinated in Petri dishes lined with filter paper moistened with an 80 mmol/L mixed saline alkali solution (Na_2_CO_3_:NaHCO_3_ = 1:9 molar ratio) and kept in a light incubator (BSG-250, Boxun, Shanghai, China ) at 25 ± 1 °C with a 16 h photoperiod. Treatments used were as follows: SS (saline, non-inoculated); SS + B1 (saline, inoculated with DP25); SS + B2 (saline, inoculated with DP28); SS + B3 (saline, inoculated with DP29); SS + Com (saline inoculated with a consortium of DP25, DP28, and DP29). Each treatment consisted of four replicates with 400 seeds, and germination was assessed over a 7-day period. Germination was recorded daily, and seeds were considered germinated when the radicle exceeded 2 mm. Germination indices, including germination rate, germination potential, germination index, and vigor index, were calculated following the method of Deng et al. [42].

### 4.4. Pot Experiment Design

The pot experiment was conducted at Shanxi Agricultural University, China (37°42′ N, 112°56′ E). The growth substrate consisted of a peat–vermiculite mixture (2:1, *v*/*v*). Before sowing, a mixed saline–alkali solution (Na_2_CO_3_:NaHCO_3_ = 1:9 molar ratio) was incorporated at a 1:1 ratio (*v*/*w*) relative to the substrate mass, while sterilized distilled water served as the control. After drying, each pot (25 × 18 × 20 cm) was filled with 500 g of substrate, into which 20 seeds were sown. Each treatment was replicated with four pots. Seedling emergence was monitored daily for 15 days, after which, seedlings were thinned to six per pot. As a whole, there were three treatments: (1) CK: substrate without saline–alkali solution, and seeds coated without any substance; (2) SS: substrate with saline–alkali solution, and seeds coated without any substance; (3) SS + Com: the substrate with saline–alkali solution, and seeds coated with bacterial consortium inoculant. Watering was adjusted every three days by the weighing method to maintain uniform substrate moisture. The total growth period was 45 days.

### 4.5. Plant Growth Parameter Analysis

At harvest, twelve seedlings per treatment were randomly selected. Plant height and root length were measured with a ruler, and shoot and root dry weights were determined using an electronic balance (ME104E, Mettler Toledo, Greifensee, Switzerland). Samples were oven-dried at 105 °C for 30 min, followed by drying at 65 °C to a constant weight.

Root systems were scanned with a desktop root scanner (V850 pro, EPSON, Tokyo, Japan), and root traits (total surface area, average diameter and volume) were quantified using Win RHIZO 3.1 software (Regent Instruments Inc., Québec, QC, Canada).

### 4.6. Determination of Photosynthetic Parameters and Photosynthetic Pigment

Photosynthetic gas exchange parameters, including net photosynthetic rate (Pn), stomatal conductance (Gs), transpiration rate (Tr), and intercellular CO_2_ concentration (Ci), were measured on the third fully expanded leaf with a portable photosynthesis system (Li-6800 or Li-6400XT, LI-COR Inc., Lincoln, NE, USA) between 9:00 and 11:00.

Chlorophyll content was quantified by the ethanol extraction method [43]. Briefly, fresh leaf tissue (0.05 g) was immersed in 15 mL of 95% ethanol and kept in darkness until pigments were fully extracted. Absorbance was then measured spectrophotometrically at 665 nm (chlorophyll a, Chl a), 649 nm (chlorophyll b, Chl b), and 470 nm (carotenoids, Carot).

### 4.7. Determination of Antioxidant Enzyme Activities, Malondialdehyde (MDA), and Osmoprotectant Levels

The levels of superoxide dismutase (SOD), peroxidase (POD), catalase (CAT), and malondialdehyde (MDA) in leaves were assayed using the NBT photoreduction, guaiacol colorimetric, UV absorption, and thiobarbituric acid (TBA) methods, respectively [44]. In brief, 0.3 g of freshly harvested leaves was homogenized in 5 mL of ice-cold 50 mmol/L sodium phosphate buffer (pH 7.8) containing 1 mmol/L EDTA·Na_2_ and 2% (*w*/*v*) polyvinylpyrrolidone (PVP). The homogenate was centrifuged at 12,000× *g* for 15 min at 4 °C, and the supernatant was collected as the crude enzyme extract for subsequent antioxidant enzyme activity and MDA determination.

Soluble sugar was determined following the anthrone–sulfuric acid method, while soluble protein was measured using the Bradford assay. Proline was quantified by the acid–ninhydrin method. Specifically, leaf tissues were extracted with 80% ethanol and reacted with anthrone reagent, and absorbance was measured at 620 nm to determine soluble sugar content. For protein, samples were incubated with Bradford dye reagent at room temperature for 10 min, and absorbance was recorded at 595 nm. Free proline was extracted with 3% (*w*/*v*) sulfosalicylic acid, reacted with acidic ninhydrin (acetic acid/phosphoric acid) at 100 °C for 60 min, cooled in an ice bath, and extracted with toluene, and the absorbance of the toluene phase was measured at 520 nm [45].

### 4.8. Assessment of Soil Properties

The rhizosphere soil samples were obtained from the 24 seedlings after harvest, pooled, passed through a 2 mm sieve, and air-dried for subsequent soil property analysis. A handheld pH meter (PHS-3E, INESA Scientific Instrument Co., Ltd., Shanghai, China) and a conductivity meter (DDS-307A, INESA Scientific Instrument Co., Ltd., Shanghai, China) were employed to determine soil pH and EC values at a soil–water ratio of 1:2 (*w*/*v*). Total nitrogen (TN) and available nitrogen (AN) were analyzed using the micro-Kjeldahl and alkaline dispersion methods, respectively [46]. Total phosphorus (TP) and available phosphorus (AP) were quantified following the procedure of Fu et al. [47]. Soil organic matter was determined by the potassium dichromate oxidation method [48].

Soil enzyme activities were determined as follows: urease, β-glucosidase, catalase, and alkaline phosphatase activities using the procedures of Yu et al. [49]; sucrase activity according to Xu et al. [50].

### 4.9. Data Statistics and Analysis

Data were organized using Microsoft Excel 2021 (Microsoft Corp., Redmond, WA, USA). Statistical analyses were performed in SPSS version 27.0 (IBM SPSS Inc., Chicago, IL, USA), including one-way analysis of variance (ANOVA) followed by Duncan’s multiple range test for significance determination (*p* < 0.05). Figures were prepared with OriginPro 2024 (OriginLab Inc., Northampton, MA, USA).

## 5. Conclusions

This study revealed that co-inoculation with the bacterial consortium including *Acinetobacter calcoaceticus* DP25, *Staphylococcus epidermidis* DP28 and *Enterobacter hormaechei* DP29 markedly promoted alfalfa growth and physiological performance under saline–alkali stress. The combined inoculation improved seed germination, emergence rate, and biomass accumulation, while alleviating stress-induced declines in photosynthetic efficiency, chlorophyll synthesis, antioxidant defense, and osmolyte accumulation. Furthermore, inoculation improved soil quality by lowering pH and EC and by increasing nutrient availability and enzymatic activities under saline–alkali conditions. Such changes were strongly correlated with improved root system architecture and overall plant performance. Therefore, the use of these microbial inoculants (DP25, DP28, and DP29) could be considered a promising strategy to enhance alfalfa establishment and growth under saline–alkali stress and to aid in the remediation of degraded saline–alkali soils. In the future, we should also focus on conducting field trials to confirm the efficacy of these inoculants across diverse environments and on molecular-level investigations to elucidate the mechanisms governing plant–microbe–soil interactions, thereby supporting their wider agricultural application.

## Figures and Tables

**Table 1:** Effects of bacterial consortium on alfalfa seed germination under saline–alkali stress.

Treatment	Germination Rate (%)	Germination Potential (%)	Germination Index	Vigor Index
SS	52.75 ± 1.14 c	37.40 ± 3.03 d	13.01 ± 2.35 c	286.50 ± 1.71 c
SS + B1	68.25 ± 2.48 b	46.40 ± 2.21 c	16.60 ± 1.62 b	359.49 ± 3.32 b
SS + B2	71.50 ± 3.49 b	45.20 ± 1.57 c	16.33 ± 1.75 b	364.87 ± 4.26 b
SS + B3	73.75 ± 1.85 b	53.60 ± 1.86 b	17.74 ± 3.30 b	360.14 ± 2.22 b
SS + Com	79.75 ± 1.72 a	68.60 ± 3.13 a	27.78 ± 1.68 a	417.49 ± 1.32 a

SS: saline–alkali stress; SS + B1: saline–alkali stress + DP25; SS + B2: saline–alkali stress + DP28; SS + B3: saline–alkali stress + DP29; SS + Com: saline–alkali stress + combination. Different letters indicate significant differences (*p* < 0.05).

## Data Availability

The original contributions presented in this study are included in the article. Further inquiries can be directed to the corresponding author.

## References

[1] Khoso M.A., Wang M., Zhou Z., Huang Y., Li S., Zhang Y., Qian G., Ko S.N., Pang Q., Liu C. (2024). *Bacillus altitudinis* AD13-4 Enhances Saline-Alkali Stress Tolerance of Alfalfa and Affects Composition of Rhizosphere Soil Microbial Community. Int. J. Mol. Sci..

[2] Guo K., Xu Z., Huo Y., Sun Q., Wang Y., Che Y., Wang J., Li W., Zhang H. (2020). Effects of Salt Concentration, pH, and Their Interaction on Plant Growth, Nutrient Uptake, and Photochemistry of Alfalfa (*Medicago sativa*) Leaves. Plant Signal. Behav..

[3] Fang S., Hou X., Liang X. (2021). Response Mechanisms of Plants under Saline-Alkali Stress. Front. Plant Sci..

[4] Sun Y., Tang L., Cui Y., Yang D., Gao H., Chen J., Zheng Z., Guo C. (2025). Inoculation of Plant Growth-Promoting Rhizobacteria and Rhizobia Changes the Protist Community of Alfalfa Rhizosphere Soil under Saline-Alkali Environment. Appl. Soil Ecol..

[5] De Andrade L.A., Santos C.H.B., Frezarin E.T., Sales L.R., Rigobelo E.C. (2023). Plant Growth-Promoting Rhizobacteria for Sustainable Agricultural Production. Microorganisms.

[6] Li Y., Zhang J., Wang X., Feng Z., Yang E., Wu M., Jiang Y., Huang J., Gao Z., Du Y. (2025). The Synergistic Effect of Extracellular Polysaccharide-Producing Salt-Tolerant Bacteria and Biochar Promotes Grape Growth under Saline-Alkaline Stress. Environ. Technol. Innov..

[7] De Oliveira L.M., Kavamura V.N., Clark I.M., Mauchline T.H., Desouza J.T. (2024). Diversity and Multifunctional Potential for Plant Growth Promotion in Bacteria from Soil and the Rhizosphere. Soil Use Manag..

[8] Khalilpour M., Mozafari V., Abbaszadeh-Dahaji P. (2021). Tolerance to Salinity and Drought Stresses in Pistachio (*Pistacia vera* L.) Seedlings Inoculated with Indigenous Stress-Tolerant PGPR Isolates. Sci. Hortic..

[9] Paul S., Parvez S.S., Goswami A., Banik A. (2024). Exopolysaccharides from Agriculturally Important Microorganisms: Conferring Soil Nutrient Status and Plant Health. Int. J. Biol. Macromol..

[10] Han L., Zhang M., Du L., Zhang L., Li B. (2022). Effects of *Bacillus amyloliquefaciens* QST713 on Photosynthesis and Antioxidant Characteristics of Alfalfa (*Medicago sativa* L.) under Drought Stress. Agronomy.

[11] Song J., Guan X., Chen L., Han Z., Cui H., Ma S. (2025). Cooperative Interplay between PGPR and *Trichoderma longibrachiatum* Reprograms the Rhizosphere Microecology for Improved Saline Alkaline Stress Resilience in Rice Seedlings. Microorganisms.

[12] Sarsan S., Pandiyan A., Rodhe A.V., Jagavati S., Singh R.P., Manchanda G., Bhattacharjee K., Panosyan H. (2021). Synergistic Interactions among Microbial Communities. Microbes in Microbial Communities: Ecological and Applied Perspectives.

[13] Gao H., Yang D., Yang L., Han S., Liu G., Tang L., Chen J., Wang D., Guo C. (2023). Co-Inoculation with *Sinorhizobium meliloti* and *Enterobacter ludwigii* Improves the Yield, Nodulation, and Quality of Alfalfa (*Medicago sativa* L.) under Saline-Alkali Environments. Ind. Crops Prod..

[14] Yuan L., Wu Y., Fan Q., Li P., Liang J., Liu Y., Ma R., Li R., Shi L. (2023). Remediating Petroleum Hydrocarbons in Highly Saline-Alkali Soils Using Three Native Plant Species. J. Environ. Manag..

[15] Guo L., Zhang X., Liu Y., Zhang A., Song W., Li L., Zhao J., Pang Q. (2025). Salt-Alkali-Tolerant Growth-Promoting *Streptomyces* sp. Jrh8-9 Enhances Alfalfa Growth and Resilience under Saline-Alkali Stress through Integrated Modulation of Photosynthesis, Antioxidant Defense, and Hormone Signaling. Microbiol. Res..

[16] Yang D., Tang L., Chen J., Shi Y., Zhou H., Gao H., Jin J., Guo C. (2024). Strategy of Endophytic Bacterial Communities in Alfalfa Roots for Enhancing Plant Resilience to Saline–Alkali Stress and Its Application. Biol. Fertil. Soils.

[17] Ling L., An Y., Wang D., Tang L., Du B., Shu Y., Bai Y., Guo C. (2022). Proteomic Analysis Reveals Responsive Mechanisms for Saline-Alkali Stress in Alfalfa. Plant Physiol. Biochem..

[18] Bal H.B., Nayak L., Das S., Adhya T.K. (2013). Isolation of ACC Deaminase Producing PGPR from Rice Rhizosphere and Evaluating Their Plant Growth Promoting Activity under Salt Stress. Plant Soil.

[19] Zhang L., Zhang M., Huang S., Li L., Gao Q., Wang Y., Zhang S., Huang S., Yuan L., Wen Y. (2022). A Highly Conserved Core Bacterial Microbiota with Nitrogen-Fixation Capacity Inhabits the Xylem Sap in Maize Plants. Nat. Commun..

[20] Damodaran T., Jha S.K., Kumari S., Gupta G., Mishra V.K., Sharma P.C., Gopal R., Singh A., Jat H.S. (2023). Development of Halotolerant Microbial Consortia for Salt Stress Mitigation and Sustainable Tomato Production in Sodic Soils: An Enzyme Mechanism Approach. Sustainability.

[21] Han Z., Chen L., Wang W., Guan X., Song J., Ma S. (2024). Biochemical and Transcriptomic Analyses Reveal Key Salinity and Alkalinity Stress Response and Tolerance Pathways in *Salix linearistipularis* Inoculated with *Trichoderma*. Agronomy.

[22] Chen Z., Zhang P., Wang B., Li H., Li S., Zhang H., Haider F.U., Li X. (2025). Harnessing the Role of Rhizo-Bacteria to Mitigate Salinity Stress in Rice (*Orzya sativa*); Focus on Antioxidant Defense System, Photosynthesis Response, and Rhizosphere Microbial Diversity. Rhizosphere.

[23] Li Y., He N., Hou J., Xu L., Liu C., Zhang J., Wang Q., Zhang X., Wu X. (2018). Factors Influencing Leaf Chlorophyll Content in Natural Forests at the Biome Scale. Front. Ecol. Evol..

[24] Yu M., Wu Q., Zheng D., Feng N., Liang X., Liu M., Li Y., Mou B. (2022). Plant Growth Regulators Enhance Saline-Alkali Tolerance by Upregulating the Levels of Antioxidants and Osmolytes in Soybean Seedlings. J. Plant Growth Regul..

[25] Li G., Shi M., Wan W., Wang Z., Ji S., Yang F., Jin S., Zhang J. (2024). Maize Endophytic Plant Growth-Promoting Bacteria *Peribacillus Simplex* Can Alleviate Plant Saline and Alkaline Stress. Int. J. Mol. Sci..

[26] Xian X., Zhang Z., Wang S., Cheng J., Gao Y., Ma N., Li C., Wang Y. (2024). Exogenous Melatonin Strengthens Saline-Alkali Stress Tolerance in Apple Rootstock M9-T337 Seedlings by Initiating a Variety of Physiological and Biochemical Pathways. Chem. Biol. Technol. Agric..

[27] Bai J., Liu J., Zhang N., Yang J., Sa R., Wu L. (2013). Effect of Alkali Stress on Soluble Sugar, Antioxidant Enzymes and Yield of Oat. J. Integr. Agric..

[28] Irigoyen J.J., Einerich D.W., Sánchez-Díaz M. (1992). Water Stress Induced Changes in Concentrations of Proline and Total Soluble Sugars in Nodulated Alfalfa (*Medicago sativa*) Plants. Physiol. Plant..

[29] Gandonou C.B., Bada F., Abrini J., Skali-Senhaji N. (2011). Free Proline, Soluble Sugars and Soluble Proteins Concentration as Affected by Salt Stress in Two Sugarcane (*Saccharum* sp.) Cultivars Differing in Their Salt Tolerance. Int. J. Mol. Sci..

[30] Moustakas M., Sperdouli I., Kouna T., Antonopoulou C.I., Therios I. (2011). Exogenous Proline Induces Soluble Sugar Accumulation and Alleviates Drought Stress Effects on Photosystem II Functioning of *Arabidopsis Thaliana* Leaves. Plant Growth Regul..

[31] Bisht N., Singh T., Ansari M.M., Joshi H., Mishra S.K., Chauhan P.S. (2024). Plant Growth-Promoting *Bacillus amyloliquefaciens* Orchestrate Homeostasis under Nutrient Deficiency Exacerbated Drought and Salinity Stress in *Oryza sativa* L. Seedlings. Planta.

[32] Zhao Y., Zhang F., Mickan B., Wang D., Wang W. (2022). Physiological, Proteomic, and Metabolomic Analysis Provide Insights into *Bacillus* Sp.-Mediated Salt Tolerance in Wheat. Plant Cell Rep..

[33] Ali B., Wang X., Saleem M.H., Sumaira, Hafeez A., Afridi M.S., Khan S., Zaib-Un-Nisa, Ullah I., Amaral Júnior A.T.D. (2022). PGPR-Mediated Salt Tolerance in Maize by Modulating Plant Physiology, Antioxidant Defense, Compatible Solutes Accumulation and Bio-Surfactant Producing Genes. Plants.

[34] Wei H., He W., Li Z., Ge L., Zhang J., Liu T. (2022). Salt-Tolerant Endophytic Bacterium *Enterobacter ludwigii* B30 Enhance Bermudagrass Growth under Salt Stress by Modulating Plant Physiology and Changing Rhizosphere and Root Bacterial Community. Front. Plant Sci..

[35] Yan N., Wang W., Mi T., Zhang X., Li X., Du G. (2024). Enhancing Tomato Growth and Soil Fertility under Salinity Stress Using Halotolerant Plant Growth-Promoting Rhizobacteria. Plant Stress.

[36] Kumar M., Yusuf M.A., Chauhan P.S., Nigam M. (2017). *Pseudomonas putida* and *Bacillus amyloliquefaciens* Alleviates the Adverse Effect of Pesticides and Poise Soil Enzymes Activities in Chickpea (*Cicer arietinum* L.) Rhizosphere. Trop. Plant Res..

[37] Hidri R., Mahmoud O.M.-B., Zorrig W., Mahmoudi H., Smaoui A., Abdelly C., Azcon R., Debez A. (2022). Plant Growth-Promoting Rhizobacteria Alleviate High Salinity Impact on the Halophyte *Suaeda fruticosa* by Modulating Antioxidant Defense and Soil Biological Activity. Front. Plant Sci..

[38] Han L., Su D., Lv S., Luo Y., Li X., Jiao J., Diao Z., Bu H. (2017). Responses of Biogeochemical Characteristics and Enzyme Activities in Sediment to Climate Warming under a Simulation Experiment in Geographically Isolated Wetlands of the Hulunbuir Grassland, China. Int. J. Environ. Res. Public Health.

[39] Li C., Jia Z., Zhai L., Zhang B., Peng X., Liu X., Zhang J. (2021). Effects of Mineral-Solubilizing Microorganisms on Root Growth, Soil Nutrient Content, and Enzyme Activities in the Rhizosphere Soil of *Robinia pseudoacacia*. Forests.

[40] Qiao Y., Wang Z., Sun H., Guo H., Song Y., Zhang H., Ruan Y., Xu Q., Huang Q., Shen Q. (2024). Synthetic Community Derived from Grafted Watermelon Rhizosphere Provides Protection for Ungrafted Watermelon against *Fusarium oxysporum* Via Microbial Synergistic Effects. Microbiome.

[41] Kossalbayev B.D., Wei M., Wang J., Pang Y., Lv M., Sadvakasova A.K., Bauenova M.O., Zhang X., Zhao W., Xu S. (2025). Growth Promotion of Synthetic Microbial Communities Influenced by the Function, Diversity and Interactions of Their Constituent Strains and Soil Types. World J. Microbiol. Biotechnol..

[42] Deng Y., Yuan F., Feng Z., Ding T., Song J., Wang B. (2014). Comparative Study on Seed Germination Characteristics of Two Species of Australia Saltbush under Salt Stress. Acta Ecol. Sin..

[43] Lichtenthaler H.K., Wellburn A.R. (1983). Determinations of Total Carotenoids and Chlorophylls a and b of Leaf Extracts in Different Solvents. Biochem. Soc. Trans..

[44] Basit F., Chen M., Ahmed T., Shahid M., Noman M., Liu J., An J., Hashem A., Fahad Al-Arjani A.B., Alqarawi A.A. (2021). Seed Priming with Brassinosteroids Alleviates Chromium Stress in Rice Cultivars Via Improving ROS Metabolism and Antioxidant Defense Response at Biochemical and Molecular Levels. Antioxidants.

[45] Abdel Latef A., Sallam M. (2015). Changes in Growth and Some Biochemical Parameters of Maize Plants Irrigated with Sewage Water. Austin J. Plant Biol..

[46] Kirk P.L. (1950). Kjeldahl Method for Total Nitrogen. Anal. Chem..

[47] Fu H., Li H., Yin P., Mei H., Li J., Zhou P., Wang Y., Ma Q., Jeyaraj A., Thangaraj K. (2021). Integrated Application of Rapeseed Cake and Green Manure Enhances Soil Nutrients and Microbial Communities in Tea Garden Soil. Sustainability.

[48] Li C., Liu L., Guo B., Zhao J., Ren Y. (2017). Potassium Dichromate Oxidation Methods’ Review and Application for Determination of Soil Organic Matter. Metall. Res. Technol..

[49] Yu H., Si P., Shao W., Qiao X., Yang X., Gao D., Wang Z. (2016). Response of Enzyme Activities and Microbial Communities to Soil Amendment with Sugar Alcohols. Microbiologyopen.

[50] Xu R., Li J., Li X., Zhang J., Song W. (2024). Effect of Coal Mining Subsidence on Soil Enzyme Activity in Mining Areas with High Underground Water Levels. Water.

